# Cell-matrix reciprocity in 3D culture models with nonlinear elasticity

**DOI:** 10.1016/j.bioactmat.2021.08.002

**Published:** 2021-08-14

**Authors:** Kaizheng Liu, Maury Wiendels, Hongbo Yuan, Changshun Ruan, Paul H.J. Kouwer

**Affiliations:** a Research Center for Human Tissue and Organs Degeneration, Institute of Biomedicine and Biotechnology, Shenzhen Institutes of Advanced Technology, Chinese Academy of Sciences, Shenzhen, 518055, PR China; b Radboud University, Institute for Molecules and Materials, Heyendaalseweg 135, 6525 AJ, Nijmegen, the Netherlands; c Institute of Biophysics, Hebei University of Technology, Tianjin, 300401, PR China; d Molecular Imaging and Photonics, Chemistry Department, KU Leuven, Celestijnenlaan 200F, 3001, Heverlee, Belgium

**Keywords:** Artificial extracellular matrices, Mechanical reciprocity, Collagen, Fibrin, Polyisocyanides

## Abstract

Three-dimensional (3D) matrix models using hydrogels are powerful tools to understand and predict cell behavior. The interactions between the cell and its matrix, however is highly complex: the matrix has a profound effect on basic cell functions but simultaneously, cells are able to actively manipulate the matrix properties. This (mechano)reciprocity between cells and the extracellular matrix (ECM) is central in regulating tissue functions and it is fundamentally important to broadly consider the biomechanical properties of the *in vivo* ECM when designing *in vitro* matrix models. This manuscript discusses two commonly used biopolymer networks, i.e. collagen and fibrin gels, and one synthetic polymer network, polyisocyanide gel (PIC), which all possess the characteristic nonlinear mechanics in the biological stress regime. We start from the structure of the materials, then address the uses, advantages, and limitations of each material, to provide a guideline for tissue engineers and biophysicists in utilizing current materials and also designing new materials for 3D cell culture purposes.

## Highlights

• Fibrous matrices closely resemble the architecture of tissues.
• Key examples include collagen, fibrin and the synthetic matrix PIC.
• Reciprocity between cells and matrix gives rise to complex behavior.
• Synthetic designer matrices give the opportunity to customize individual parameters.

## Graphical abstract

**Figure:**
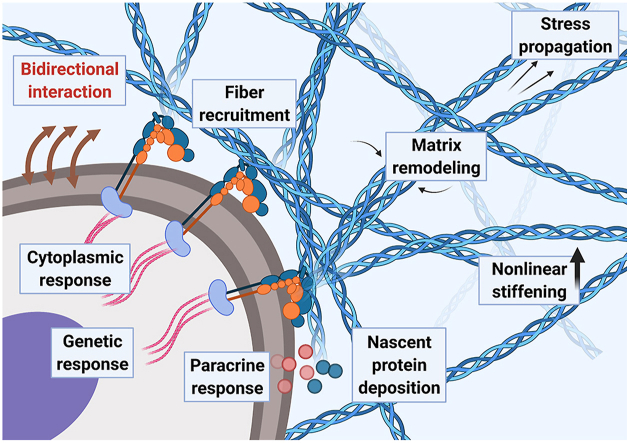

## 1 The role of matrix mechanics in *in vivo* microenvironments

Cells *in vivo* reside in a highly dynamic and complex microenvironment composed of a plethora of interacting materials termed the extracellular matrix (ECM) [1]. The ECM provides mechanical support for tissues and organs and initiates or facilitates biochemical and biomechanical signals, which are required for morphogenesis, homeostasis, and tissue repair [2,3]. The ECM should be considered a natural hydrogel composed of a network of fibrous proteins such as fibronectin, collagen, fibrin, laminin, and elastin, which are accompanied by hydrated (nonfibrous) proteoglycans and polysaccharides, such as hyaluronic acid [4]. From an architectural point of view, the relatively large (micrometer scales) pores of the fibrous network allow facile transport of metabolic molecules between cells [5]; the distribution and anisotropy of fibers impose a crucial impact on the tissues as well [6]. It is well established that the fibrous ECM proteins are the main responsible components for mechanical interactions with cells [7].

At the cellular level, the ECM is a key regulator for many cellular functions and processes, including (stem cell) differentiation, migration, growth, and survival [8,9]. Its mechanical properties, chemical composition, and structural organization induce a variety of signaling pathways inside residing cells [10]. Transfer of mechanical information follows a process named mechanotransduction [11,12], which is mediated by molecular linkages at the cell-matrix interface, called focal adhesions [3,13]. For the molecular cell-matrix interactions, cells express a range of integrins, proteins that bind various components of the ECM. The adhesion complexes assemble and mature in a force-dependent manner, starting as very small nascent adhesions and developing into large focal adhesions in a timespan as short as seconds to minutes (at least on the planar surface) [14,15]. The physical properties of the ECM are transmitted from the fibrous ECM components through the adhesion complexes to the cytoskeleton and then further intracellularly processed into biochemical signals [15,16]. We note that most established mechanisms of cell mechanosensing are based on 2D cell cultures (for detailed reviews, please read Miller et al. [17] and Jansen et al. [11]). Currently, we still have limited knowledge about the process in 3D [11,18], which closer mimics the *in vivo* conditions; some (recent) reviews discuss the progress made over the last decade [[19], [20], [21], [22]]. Here, we briefly highlight key aspects that one should consider by changing the cell culture experiment from 2D to 3D: On the matrix's side, the increase in matrix dimensionality results in mechanical confinement for embedded cells. In addition, the nature, and the time and length scales of the (transient) hierarchical fibrous architecture of matrix material make the 3D microenvironment more complex. Consequently, on the cells' side, disparate adhesions and adhesion kinetics are formed, and alternative signaling pathways are activated [23,24]. Furthermore, cells show different phenotypes and migration modes compared to the counterparts grown on 2D planar surfaces.

In turn, cells are able to actively manipulate their ECM. They exert tension onto the ECM fibers and affect their own fate. At longer time scales cells digest the ECM or deposit additional ECM components, basically changing the concentration of (specific) biopolymers, and, consequently, the properties of their microenvironment. At very short time scales the matrix gives an immediate stiffening response as a result of contractile forces applied by the cell. The reciprocal interaction between the ECM and the residing cell makes the field interesting and attractive, but at the same time quite challenging to study.

Stiffness, as the classical physical parameter, together with other ECM properties, plays an important role in regulating cell behaviors in both physiological and pathological contexts [25]. In nature, the stiffness of tissues is tightly controlled [26]; changes in tissue or organ stiffness are associated with diseases such as cancer and fibrosis [27]. Numerous *in vivo* cell culture studies have uncovered the potential influences of matrix stiffness on cell functions such as cell proliferation and differentiation, migration, morphology, the degree of the cell-ECM adhesions, and the size of (focal) adhesions, both in 2D [25,28,29] and 3D [20,30] studies. It is important to realize, however, that what is generally considered the stiffness is often the storage modulus of the corresponding cell-void matrix, determined at small strains during a rheology experiment [31]. For most traditional synthetic polymer matrices, a rheology measurement represents the stiffness well, but for many biological materials, the stiffness is not a static value. For biological matrices, cellular contractility imposes stress on the gel, which, as a result of its stress-stiffening characteristics (Box 1) can become many times stiffer [32,33]. As these stresses are locally applied, stiffening is highly non-uniform, i.e., the effect is strongest close to the cells, although cell contraction-induced matrix stiffening can even be measured macroscopically [34]. Note that in this case, two mechanisms of stiffening function simultaneously, one is the stiffening from the nonlinearity of the stress-strain curve, and a second one is the stiffening due to an increase in polymer concentration in the vicinity of the cell.

Box 1 **The nonlinear mechanical properties of biological gels**

It is widely acknowledged that tissue mechanics plays a vital role in regulating the biological functions of the human body. Understanding the fundaments of cell-matrix mechanobiology [40] will accelerate progress in tissue engineering and associated fields. *Ex vivo* experiments reveal that biological materials from soft tissues (e.g. blood vessels, mesentery tissue, lung parenchyma, and cornea) [32] and hard tissues (e.g. cortical bones [41]) stiffen under (shear) strain. Mechanical analysis of many reconstituted gels of structural biopolymers, such as type I collagen and fibrin (as well as the cytoskeletal F-actin and intermediate filaments) show a similar stiffening behavior. At small deformations, these fibrous hydrogels display a constant stiffness or storage modulus *G*′, which is readily tuned by changing the polymer concentration, or sometimes, by tailoring the preparation conditions. When, however, an internal or external stress (or strain) is applied that exceeds a critical value, the biological gels show a strong nonlinear stiffening response (also termed strain stiffening or stress stiffening), which could reach hundreds of times the value of the original low-stress modulus [32]. Interestingly, under such strain, the stiffness of these hydrogels has become independent of the protein concentration [3,9]. Note that in this review, we particularly focus on strain-induced stiffening as a nonlinear mechanical effect. Other nonlinear effects, such as strain-softening or compression softening are not included.

From shear rheology experiments in the linear viscoelastic (LVE) regime, one obtains the storage modulus *G*′ = *σ*/*γ* as the ratio between the stress *σ* and strain *γ*. For predominantly elastic gels that show a modulus that is independent of the deformation frequency, often the term plateau modulus *G*_0_ is used for *G*′. At increased stress [42], the material stiffens and in this nonlinear regime, the modulus is more accurately described by the differential modulus *K*′*=* ∂*σ*/∂*γ* (note that in the LVE regime, *K*′ *= G*′). For these fibrous networks, the full mechanical properties need to be described by three parameters: (*i*) the storage modulus at low stress or strain (in the LVE regime); (*ii*) the point at which stress the gels start to show the nonlinear strain response, defined as the critical stress *σ*_c_ or critical strain *γ*_c_: and (*iii*) the extent of stiffening, expressed as the stiffening index *m* that is quantified by fitting the power law *K*′ ~ *σ*^*m*^. The metrics *σ*_c_ (or *γ*_c_) and *m* can be considered the sensitivity and responsiveness of a hydrogel: a low *σ*_c_ characterizes a highly stress-responsive gel and a high *m* describes a strong response. The nonlinear mechanical terms depend on many network parameters, including concentration, polymer characteristics such as chain length and persistence length and fibril formation and are influenced by environmental conditions, like temperature and ionic strength [43]. The debate on the biological functions of the nonlinear mechanical properties of the ECM is still ongoing [9], but certainly, it is believed to be involved in the structural integrity of tissues [32] and cell-cell communication [3].

Two mechanisms have been reported that account for the nonlinear mechanics of semi-flexible polymer networks [44,45]. The first is the entropic model, which considers affine deformations, i.e. local microscopic strains exactly follow the global macroscopic deformation. In this model, the filaments show thermal fluctuations that are constrained between the crosslinking points. Removal of undulations due to strain gives rise to a nonlinear force-extension relation, causing stiffening. An alternative mechanism, the enthalpic model, assumes non-affine deformations of stiff networks [46,47]. In this model, the amplitude of thermal fluctuations is negligible because of the high stiffness of the single filaments. At small strains, the filaments bend rather than stretch, while at larger strains, the filaments in the network rearrange and align towards the strain direction, marking the beginning of strain-stiffening with the transition from bending modes to stretching modes. Despite a difference in origin, both mechanisms give a similar stress-stiffening response and, since they are not mutually exclusive, both are likely to contribute to the nonlinear mechanics in heterogeneous fibrous polymer networks.

When mentioning nonlinear mechanics in this review, we consider the mechanical response of the soft material towards shear strain. Also in other geometries, nonlinear mechanical properties have been reported, for instance compression softening and extension stiffening have been observed for all three hydrogels discussed in detail in this review [[48], [49], [50]], and are considered to play a key role in cell-laden tissue mechanics [51]. In addition, recent work shows that also viscous contributions of the mechanical properties should be considered, including characteristic gel relaxation time(s) *τ* and the plasticity of the gel [37,[52], [53], [54], [55]]. To further complicate matters, the relaxation time of stress-stiffening materials typically is not constant either, but rather is a function of the applied stress [56], indicating that in these materials different stress relaxation mechanisms are active.

Although a comprehensive picture of all mechanical properties of fibrous biological gels is incredibly difficult to provide [57], it is clear that only considering the LVE ‘static’ shear modulus clearly is insufficient to capture the mechanics involving cell-gel interactions.

**Figure:**
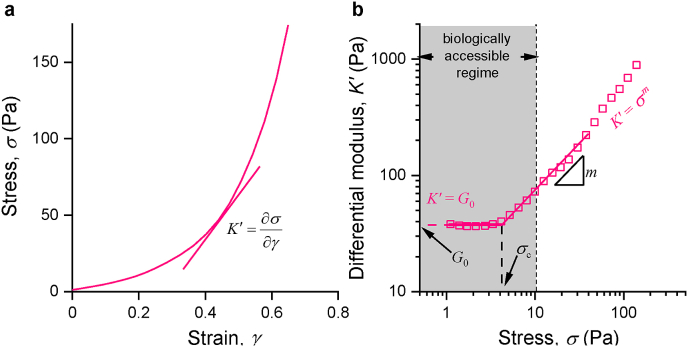

Figure Box 1. **Basic concepts in strain stiffening. a.** Stress-strain curve of a fibrous matrix in a stress ramp. **b.** The stiffness represented as the differential modulus *K′* = δ*σ*/δ*γ* as a function of stress σ for the same polymer. At low stress, *K′* = *G*_0_ the plateau modulus, but beyond a critical stress *σ*_c_, *K′* increases, following *K′* ∝ *σ*^*m*^ where the exponent *m* is the stiffening index. Figure adapted from Jasper et al. [43].

Alt-text: Box 1

We underline that stress or strain-stiffening is a unique property that is closely associated with the fibrous architecture. It is important to realize that nonlinear stiffening is not always fully reversible. Some reports propose that as soon as the (contractile) stress is removed, the stiffness reduces again [35], while other reports show that the mechanical properties of fibrous biopolymer networks (e.g. collagen [36,37] or fibrin [38,39]) depend strongly on deformation history and lack reversibility, even at small strains.

## 2 Models of the ECM to study its role in tissue functions

To understand the complex reciprocity between cells and matrices, *in vitro* ECM models have been constructed. Most studies have been carried out in traditional 2D models, where the cells are seeded on top of the gel or a scaffold. The 2D approach is well-established, experimentally very accessible, and straightforward to study with (automated) microscopy techniques. It does, however, poorly capture or mimic the 3D environment found *in vivo*. Over the past decade, 3D models are becoming increasingly popular. Here, the cells are encapsulated inside the gels, which in itself gives rise to experimental challenges of, for instance, microscopy and cell harvesting. Moreover, to manipulate the desired matrix property in a 3D-culture model, one finds that often many other parameters change simultaneously, which makes it hard to delineate the effect of a single parameter. For instance, when the concentration of a biopolymer scaffold is increased, the gel stiffness increases (intended effect), but at the same time, the average pore size decreases, stress relaxation changes, and more cell-binding sites are presented (frequently ignored ‘side’ effects). Nevertheless, the benefits of a better representation of the natural situation outweigh the experimental challenges and 3D models increasingly become the norm in the field.

The current golden standard for 3D culture matrices is based on basement membrane extracts, which simplistically, can be described as biological gels that are loaded with bioactive molecules from the natural ECM. The major advantages of these biological matrices are their intrinsic adhesiveness to cells, promising stability in culture, and biophysical properties that are similar to *in vivo* contexts [58]. Among them, Matrigel is a popular choice of product. It is a murine extract, originally developed in 1975, which forms a soft hydrogel with a network based on laminin and collagen IV that contains hundreds if not thousands of bioactive proteins and peptides [59,60]. This rich (and variable) composition, makes Matrigel often an unsuitable model to study reciprocal effects between cells and their matrix. While we include Matrigel as the standard in the overview at the summary of the manuscript, we will not discuss it in detail, mainly because the high content of the glycoprotein laminin in Matrigel alters the fibrous collagenous structure, which suppresses the typical nonlinear strain-stiffening response that is associated with the fibrous architecture [[61], [62], [63]].

Much more attractive are the better-defined biological matrices such as collagen and fibrin. In these models, the overall ECM structure is retained without the complexity of the complete ECM [64] and the matrix properties are relatively easily manipulated. A key drawback of these models is that changes in matrix stiffness often occur with changes in other material properties, such as protein concentration, pore size, ligand density, and topography [65], which makes it challenging to confidently explain experimental results. Gelatin, as the partly denatured form of collagen, is also widely applied in matrix preparation, but one should keep in mind that the denaturing process destroys the fibrous structure. Alginate, extracted from seaweeds is a popular choice of naturally derived materials with highly controllable viscoelastic mechanics [53], but its gels do not resemble the fibrous architecture of mammalian ECM.

Synthetic matrices combine the advantages of excellent chemical and architectural control with high reproducibility. Often various matrix properties can be manipulated independently, which makes them eminently suited to sort out the impact of each matrix property on the cell behavior. Various well-established materials have been developed, including gels based on polyethylene glycol (PEG), polyvinyl alcohol (PVA), polyacrylamide (PAM), and many more. We refer to the review from Annabi et al. [66] for a comprehensive overview of synthetic hydrogels for regenerative medicine. Mostly, these synthetic hydrogels are not fibrous in architecture and therefore, and they lack the intricate nonlinear mechanical properties of the biological models. In the past decade, increasingly strain-stiffening behavior is observed in fibrous synthetic or semi-synthetic materials or even in some (composite) flexible polymer-based hydrogels [[67], [68], [69], [70], [71], [72], [73]]. While the expansion in materials is good news for the field, the impact for cell culture applications in these new materials has not been developed sufficiently. So far, only polyisocyanide (PIC)-based hydrogels have established a track record as mimics of the architecture and mechanical properties of biological gels in combination with sufficient cell data. Moreover, being a synthetic material, the PIC gel properties (e.g. stiffness and pore size) can be individually tailored [74].

**Scheme 1:**
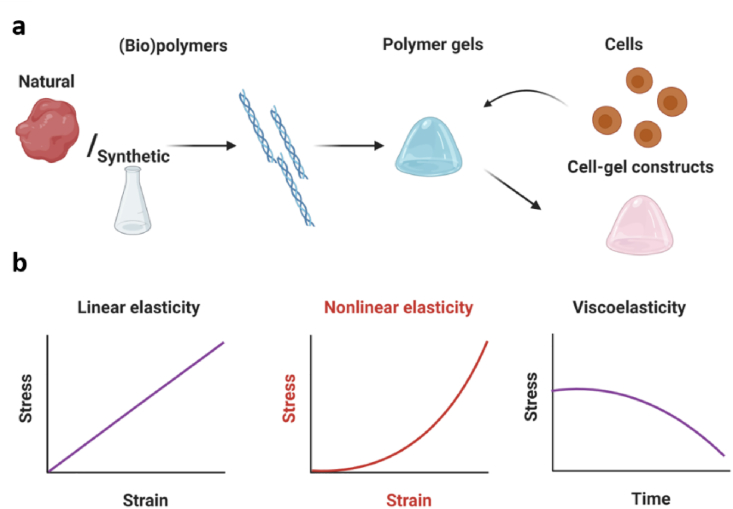
**The key parameters in mechanobiological cell-matrix studies. a.** Preparations of gel samples and cell-gel constructs. Synthetic or biological polymers are mixed with an aqueous medium (buffer, cell culture medium, etc.) to form hydrogels. To form cell-gel constructs, cells are typically introduced before the gelation sets in. **b**. The mechanical properties of the gels are conveniently measured in a rheometer. We define three major responses: gels that display simple linear mechanics without significant stiffening or relaxation (e.g. crosslinked polyacrylamide gels), gels that strain stiffen (fibrous gels discussed in this review), and gels with significant relaxation, reviewed recently [75].

The goal of this review is to discuss how mechanoreciprocity between cells and their matrix is influenced by the nonlinear mechanics of the 3D ECM. We will discuss the effects in commonly used well-defined biopolymer models (collagen and fibrin) and the synthetic polymer model based on PIC. Background on the composition and (hierarchically ordered) architecture of these materials is given in Box 2. We will cover the structures of the matrix models and compare the advantages and disadvantages of their use. Overall, these 3D *in vitro* cell culture models provide insight towards cell-matrix interactions in natural ECM from a biomechanical perspective. This information is crucial for the pioneering biologists who aim to unravel the diverse roles of the dynamic mechanics of the natural ECM. Scheme 1 summarizes the key parameters in mechanobiological cell-matrix studies, serving as a practical reference for the reader. We recommend the reviews by Broedersz et al. [92] and Alisafaei et al. [93] for readers who are interested in the theoretical modeling of fibrous networks and long-range mechanical signaling in these networks, respectively. For a perspective on the roles of biomaterial microarchitecture, please refer to Hogreben et al. [94]. We note that a few studies discussed here below are still based on 2D models due to the difficulty of performing similar experiments in 3D.

Box 2 **The composition of fibrous hydrogels**

**Fig. 1:**
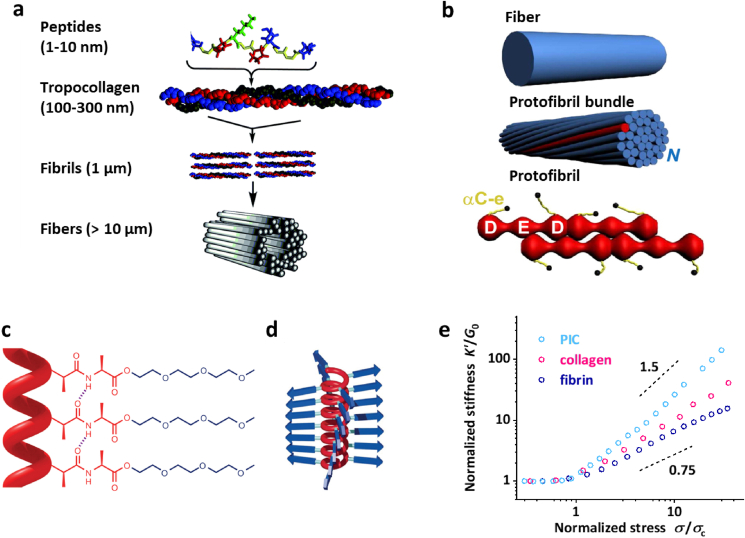
**Structure and mechanics of natural and synthetic fibrous polymer networks. a.** Hierarchical structure of collagen fibers. Figure adapted from Domene et al. [95]. **b.** Hierarchical structure of fibrin fibers. Figure adapted from Piechocka et al. [96]. **c & d.** Molecular structure and helical conformation of the PIC polymer. Figure adapted from Kouwer et al. [73]. **e.** Normalized stiffness (*K′/G*_0_) is plotted as a function of the normalized stress (*σ*/*σ*_c_) for PIC (light blue), collagen (pink), and fibrin (navy) matrices. The extent of stiffening, represented by the slope of curves in the nonlinear regimes (i.e. the stiffening index *m*) is different for each material: PIC (1.5) >collagen (1) >fibrin (0.75) [43,97,98]. Note that by changing the polymer concentration, *m* remains mostly constant for collagen and fibrin and can be varied to some extent with PIC. Reproduced with permission.

A hydrogel composed of a fibrous network offers great advantages [76]. Firstly, the architecture gives mechanical stability at low polymer concentrations; typically concentrations up to 1 %-wt are used, frequently much lower. Secondly, the fibril or bundle formation process gives rise to a highly porous architecture that can accommodate cells, without the need of (local) matrix degradation [77], and facilitates free diffusion of small and large biomolecules. Lastly, the fibrous architecture is directly responsible for the nonlinear mechanical properties. Here, we give a brief overview of the structure of the fibrous gels that we will discuss in this review (see also Fig. 1).

**Collagen.** The natural ECM consists of a heterogeneous mix of different structural proteins where one clearly dominates: collagen [1]. So far, 28 types of collagen have been identified and the majority has a structural motif in which 3 polypeptide strands coil around each other to form a right-handed triple-helix [78]. In most collagens, a perfect repeating triplet amino acid sequence of gly-X-Y can be found, where X and Y can be any amino acid [79]. Among all subtypes, type-I collagen is the most abundant protein in the family (and in the human body). The biosynthesis of collagen is a hierarchical process ranging from peptides and tropocollagen molecules at the nanometer scale to fibrils and fibers at the micrometer scale. Cells bind to collagen via α_1_β_1_ and α_2_β_1_ integrins by recognizing the characteristic peptide sequence GFOGER [80], which is also introduced in synthetic matrices mimicking collagen [15,81].

**Fibrin.** Fibrous fibrin networks support the blood clotting once a wound site occurs. Fibrin is formed by polymerizing fibrinogen, a soluble biomacromolecule, via the serine protease thrombin. Fibrinogen is a 340 kDa αβγ-chain protein formed by a dimeric glycoprotein present in human blood plasma [82]. Once the fibrin network is formed, it can be further crosslinked through the activity of transglutaminase factor XIII, supporting the positioning and stabilization of fibrin clots [83]. In addition, fibrinogen and crosslinked fibrin can be degraded by metalloproteinase 3 (MMP-3) [84]. Fibrinogen chains contain cell-adhesive RGD sequences at multiple positions that bind integrins.

**PIC hydrogels.** Oligo(ethylene glycol)-substituted polyisocyanides (PICs) are a class of synthetic rigid polymers [73]. Nickel-catalyzed polymerization of the isocyanide monomer gives rise to a helical polymer chain conformation that is stabilized by hydrogen bonding between peptide residues, usually a *d* and *l*-alanine. Variation of the nature, number, and configuration of the peptides allows tailoring the properties of the polymers and, consequently, of the hydrogels [85,86]. The length of the oligo(ethylene glycol) tail determines the gelation temperature of the aqueous polymer solution [87,88]. In the gel state, polymers are bundled into a heterogeneous network with pore sizes ranging from 0.1 to 5 μm [89].

As a synthetic polymer, cells do not adhere to PIC-based networks. For cell-PIC interactions, an azide-functionalized monomer is copolymerized (1–3%), which is converted to any desired bioactive group through the (copper-free) strain-promoted azide-alkyne cycloaddition (SPAAC) reaction [90]. By default, the commonly used generic cell-binding peptide Gly-Arg-Gly-Asp-Ser (GRGDS) is introduced. The post-modification method, however, allows for a density-controlled introduction of virtually any bioactive moiety [91].

Alt-text: Box 2

## 3 Collagen

### 3.1 About the material

Collagen is the main structural element of multiple connective tissues and is the most abundant fibrous protein in the human body. In the natural ECM, collagen is responsible for providing elastic strength and regulating cell adhesion, chemotaxis, migration, and tissue development [1], which makes it an attractive matrix material. The stiffness of reconstituted collagen hydrogels is commonly controlled by the protein concentration (and/or the preparation conditions), i.e. increasing the concentration results in a stiffer gel; the elastic shear modulus is approximately proportional to the square of protein concentration [45]. Due to the nonlinear stress-strain correlation of collagen networks, collagen-based hydrogels are suitable models to investigate the effects of a stiffening ECM.

### 3.2 Mechanoreciprocity between cells and collagen matrices

**The matrix driving cell behavior.** Numerous studies have illustrated the influence of the static matrix stiffness on cell behavior, but also the nonlinear elasticity of collagen has been reported to regulate many cell behaviors. Petrie et al. used primary human fibroblasts in 3D matrices to demonstrate the impact of matrix elasticity on the mode of cell migration [99]. They employed a microelectrode coupled to a servo-null micropressure system which penetrated the plasma membrane in front of the nucleus in the direction of the leading edge to acquire the intracellular pressure (Fig. 2a). Among two types of 3D matrices, collagen matrices with nonlinear elasticity triggered a lamellipodia-based migration mode, where low pressures were detected both in the front and at the back of the nucleus; the control matrices with linear elasticity promoted an intracellular-pressure-based mode involving lobopodia, where the pressure was compartmentalized significantly into a high and low pressure zone. (Fig. 2b). These results illustrate how cells adapt their inner biophysical properties to the (nonlinear) mechanics of the surrounding matrix.

**Fig. 2 Part I:**
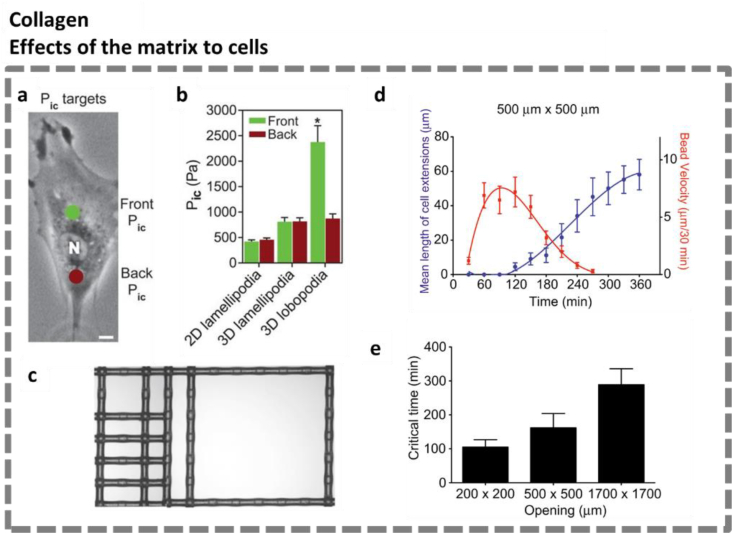
**Mechanoreciprocity between cells and collagen matrices: matrix influencing cells. a**. Intracellular pressure measurements took place in front of (green dot) and behind (red dot) the nucleus (N). Scale bar: 5 μm. **b**. Comparison of intracellular pressures in front and behind the nucleus of cells (*n* ≥ 25) migrating in different conditions (*N* = 3). *P < 0.01. Figure **a**, **b** adapted from Petrie et al. [99]. **c**. Nylon grids creating a gradient of opening sizes for cell growth (200 × 200 μm, 200 × 500 μm, 200 × 1700 μm, 500 × 500 μm, and 1700 × 1700 μm). **d**. Example illustration of the change in mean length of cell extensions (blue) and collagen compaction rate (red) as a function of time in grids with opening sizes of 500 μm wide. **e**. Critical time interval, a parameter quantifying the time for cells to fully spread out and compact collagen fibers locally, in grids of different opening widths. Cells in larger grids take longer to adapt to the microenvironment. Figure **c**, **d** & **e** adapted from Mohammadi et al. [100]. Reproduced with permission.

Nonlinear mechanics of collagen matrices also supports cells in the boundary sensing of tissues. Mohammadi et al. developed a model system that employed floating thin collagen gels surrounded by rigid grids of varying dimensions [100]. By confocal imaging, the dynamics, lengths, and number of cell extensions on top of the gels were found to be regulated by the grid size. Moreover, the cell-induced deformation fields extended to the boundaries of the smaller grids more easily, and the collagen compaction was resisted by the physical boundaries. Meanwhile, the cell-induced matrix deformation did not reach the larger grid boundaries (Fig. 2c). This research supports the notion that the fibrillar nature of collagen gels allows cells to actively sense the physical boundaries of the matrix mechanically.

**Cells affecting the matrix.** Besides the matrix affecting cell behavior, there have been a number of reports on the nonlinear mechanical response of collagen gels triggered by cells. Condor et al. investigated how cells responded to steric hindrance arising from altered cell mechanical properties, instead of gels with different polymer concentrations, to achieve better variable control [105] (Fig. 2f). They used traction force microscopy (TFM) to evaluate the mechanoreciprocity between MDA-MB 231 breast cancer cells and their surrounding collagen matrix. Overexpression of the nuclear protein lamin A or the introduction of stiff polystyrene beads inside cells were utilized to modulate cell mechanics. Their results show that the collagen matrix surrounding the cells stiffens dramatically and possesses increased strain energy, regardless of the cell mechanics. The work suggests that the matrix is stiffened by cellular contraction and can transmit deformations over large distances toward unstrained regions.

**Fig. 2 Part II:**
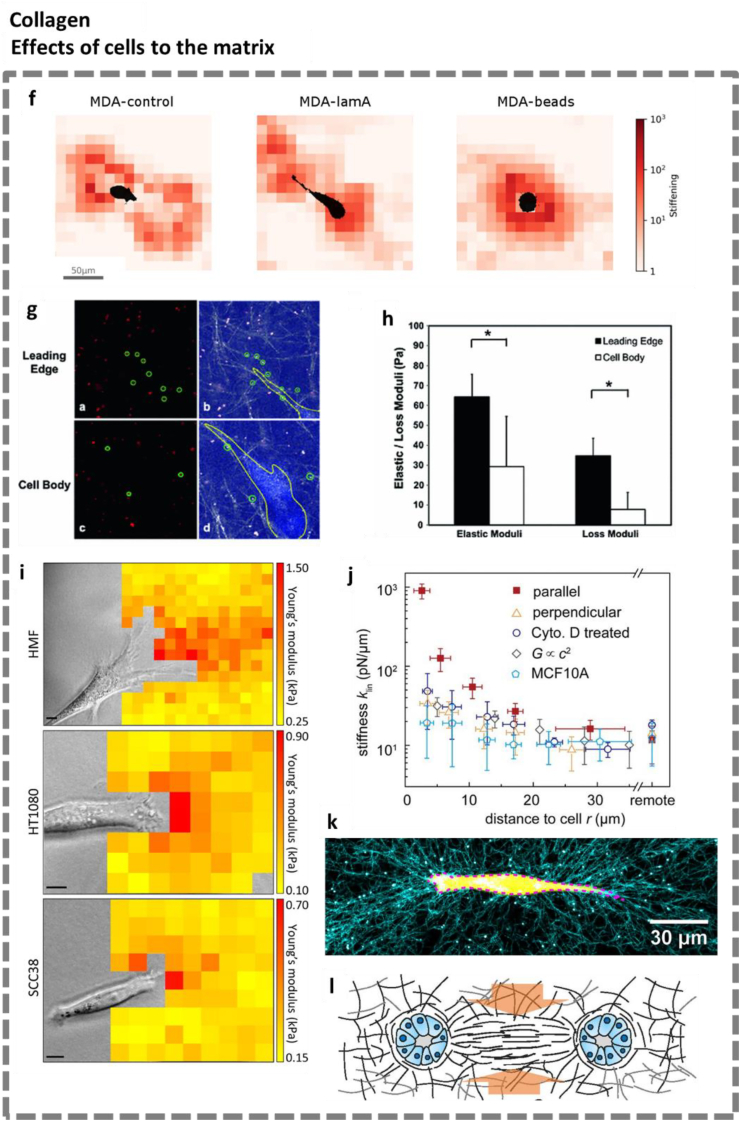
**Mechanoreciprocity between cells and collagen matrices: cells influencing the matrix. f**. Strain-stiffening maps of the collagen matrix in the vicinity of a control cell (*left*), lam-A-overexpressing cell (*middle*), and a cell with a 5-μm polystyrene bead (*right*). Figure adapted from Condor et al. [101]. **g**. Covalently bound NHS-microspheres for monitoring local stiffness at a leading edge (a, b) of a C6 glioma cell, and around the cell body (c, d) in a collagen gel. The positions of the microspheres are highlighted in green circles. **h**. Rheological data obtained by tracking the trajectories of the selected NHS-microspheres. The matrix at the leading edge of a cell was stiffer than the one surrounding the main cell body. Panel **g** & **h** adapted from Wong et al. [102]. **i**. Overlap images of the heat maps and the corresponding positions of bright-field images show that stiffness of the collagen surface is elevated adjacent to the leading edge of moving cells. Figure adapted from van Helvert et al. [103]. **j**. Quantification of the stiffness of the local 3D matrix as a function of distance to the cell at different directions and in different conditions. The stiffening of the matrix depends on the relative location to cells, cell types, and cytoskeleton functions. Red squares and yellow triangles: measurements along and perpendicular to the contraction direction of cancer cells, respectively. Blue circles: measurements along the contraction direction of cells but treated with a contraction inhibitor, cytochalasin D. Gray diamonds: stiffness expected from the increased collagen concentration *c*. Light blue polygons: measurements in the contraction direction of epithelial cells. ‘Remote’ stands for the locations that are further than 200 μm from the cell. Figure adapted from Han et al. [34]. **k**. Confocal image of a human breast adenocarcinoma cell (yellow; outlined in magenta) in a collagen gel (cyan) with 1 mg/mL collagen and 37 °C polymerization. Clear collagen fiber alignment is seen near the cell. Figure adapted from Hall et al. [104]. **l**. Schematic of the Poisson effect in a collagen tract formed by two nascent mechanically interacting cell clusters. Figure adapted from Ban et al. [48]. Reproduced with permission.

Wong et al. developed a method to measure the local network mechanics in cell-seeded collagen gels based on particle-tracking microrheology (PTM) [102] (Fig. 2g and h). Using this method, they found the local network at the leading edge of a typical C6 glioma cell to be stiffer as compared to the side. This observation indicates the stiffening of the local fiber network in collagen matrices induced by cellular contraction. Similarly, other microrheology-based work from Krajina et al. beautifully demonstrates the heterogeneous cell-mediated strain stiffening in hybrid collagen/Matrigel matrices [101].

Van Helvert et al. investigated the cell-induced stiffening of collagen matrices during cell migration [103] (Fig. 2i). The authors combined atomic force microscopy (AFM) nanoindentation and confocal microscopy to measure the strain stiffening at the leading edge of different cell types migrating across fibrillar type I collagen matrices. A gradient-like fiber realignment, densification, and elevation of Young's modulus ahead of the leading migration edge of cells were observed, and a β1 integrin- and an actomyosin-dependent mechanism was confirmed. Their work proves that the extent of matrix stiffening scales with the cell type, multicellular cooperativity, integrin availability, and contractility. Due to the working principle of the AFM, this study was performed in 2D, on top of collagen substrates.

Han et al. used nonlinear stress inference microscopy (NSIM) to identify the mechanical interactions between human breast cancer cells and collagen matrices [34]. Their results show that closer to the cells, the matrix is stiffer due to contractility-induced matrix stiffening (Fig. 2j). The increased modulus levels off further away from the cell (at an order of magnitude of 10 μm), which gives rise to stiffness gradients in the 3D collagen matrix. The modification of the mechanics of the ECM microenvironment through cell contractility is anticipated to be a crucial process for matrix-mediated interactions between cells. These data suggest that matrix stiffening induced by cells, even if they are distant, gives the ability to mediate mechanical communication between cells.

Likewise, Hall et al. confirmed that breast cancer cells exert sufficient strains to locally align and stiffen collagen matrices [104] (Fig. 2k). They observed that the cells exerted a greater strain in collagen gels with lower stiffness. The stiffness of collagen gels was controlled by varying polymer concentration, the cross-linking density, and the polymerization temperature. Using traction force microscopy (TFM), the authors found that cells in stiffer matrices generated more contractile forces and a stiffer cell body. Notably, the cell force transmission distance increased with the degree of strain-induced fiber alignment and stiffening of the collagen matrices. The work highlights the role of nonlinear mechanics of fibrous polymer networks in regulating mechanoreciprocity.

Stiffening of collagen matrices is associated with fiber alignment. In their work on vascular network formation in collagen matrices, McCoy et al. studied the influence of matrix fiber alignment by evaluating the formation of the vascular network from two different endothelial cell types (HUVECs and hCMECs) [106]. Cells encapsulated within different gels that were exposed to increasing pre-strains responded to stronger collagen fiber orientation by assembling into 3D vascular networks with thicker, more aligned branches, increased collagen IV deposition, and lumen formation compared to control conditions. The work suggests indirectly that strain stiffening of collagen matrices regulates the formation of vascular networks. Another example is the fiber alignment (also termed fiber tracts, fiber channels) observed between mechanically interacting cell clusters in collagen matrices by Ban et al. [48] (Fig. 2i). This alignment brings a contraction in the transverse direction (Poisson effect), which in turn promotes invasion of cancerous acinar cells.

## 4 Fibrin

### 4.1 About the material

Whereas collagen is the most abundant protein in the ECM, fibrin is the main component in blood clots. It is an important factor during wound healing and tissue repair in adults, albeit less during embryonic development. During tissue damage, the first response of the body is to quickly generate a fibrin seal to cover the wound. Besides the hemostatic function, fibrin is also prominently involved in wound healing by promoting physiological inflammation and angiogenesis through its interactions with leukocytes and endothelial cells [[107], [108], [109]]. Based on its role in wound healing, angiogenesis, and cell migration, fibrin is frequently used as an *in vitro* 3D cell culture scaffold [110,111]. Compared with collagen, fibrin gels are generally much more ductile and have the advantage that their mechanical properties and network architecture are tunable to a greater extent [112]. We note that at high strains, fibrin gels deform irreversibly.

### 4.2 Mechanoreciprocity between cells and fibrin matrices

**Fig. 3:**
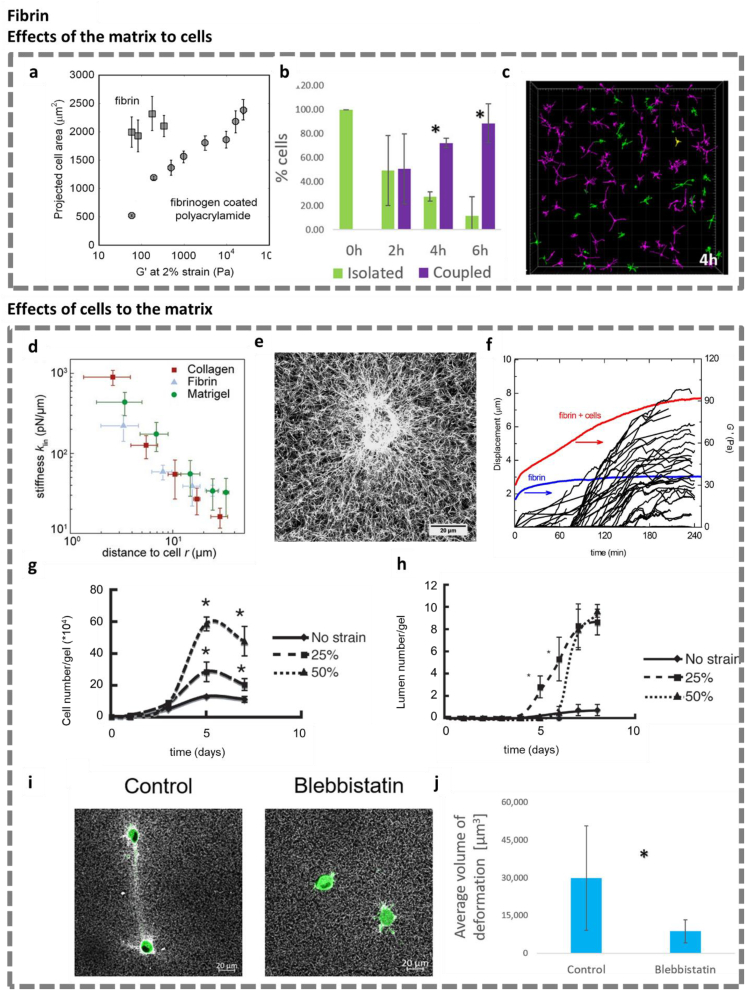
**Mechanoreciprocity between cells and fibrin matrices. a**. The spreading area of fibroblasts on fibrin and fibrinogen coated polyacrylamide gels of varying stiffness, 18 h after seeding on the substrate. Figure adapted from Winter et al. [3]. **b** & **c**. The relation between bond formation and cell morphology: (**b**) The fraction of mechanically coupled cells with time; and (**c**) the projection image of large mm-scale 3D volumes showing the coupled and isolated cells in 3D at 4 h. **d**. Local linear stiffness *k*_lin_ as a function of the distance to the cell *r* along its principal contraction direction in collagen (red squares), fibrin (blue triangles), and Matrigel (green circles). All ECM model systems exhibit a strong cell-induced stiffening gradient. Figure adapted from Han et al. [34]. **e**. Maximum intensity projection image of a cell-populated fibrin network (1 g/L), showing alignment and recruitment of fibrin fibers around the cell. **f**. Examples of the increase in traction strain (in black) and macroscopic elastic modulus of a cell-populated fibrin gel (in red) and a cell-free fibrin gel (in blue) over time. Panels **e** & **f** adapted from Jansen et al. [113]. **g** & **h**. Cell proliferation of myoblasts (**g**) and the development of lumens by HUVECs (**h**) in fibrin gels subjected to different strains. Figures adapted from Matsumoto et al. [114]. **i** & **j.** The effect of myosin II inhibition on the ability of cells to generate bands. In untreated gels, the matrix underwent dramatic deformation and many bands were formed between neighbor cells; Blebbistatin reduced matrix deformation and band formation significantly. See (**i**) for fluorescence images and (**j**) for quantification of band volumes. Panels **b**, **c**, **i** & **j** adapted from Natan et al. [115]. Reproduced with permission.

**The matrix driving cell behavior.** Winter et al. studied the role of nonlinear mechanics in cell-cell communication. They seeded fibroblasts and human mesenchymal stem cells on top of fibrin (strain-stiffening) and polyacrylamide (not stiffening) gels with different concentrations and observed the spreading of the cells [3]. Their results reveal that on the polyacrylamide gels, cell spreading increases with the gel storage modulus, but that on fibrin, the cells spread out to a maximum extent, despite the much lower stiffness of the material (Fig. 3a). In addition, the authors observed matrix deformations from several microns up to five cell lengths away from cell membranes by time-lapse microscopy. Matrix stiffening was confirmed by atomic force microscopy (local) and rheology (global). This nonlinear mechanical effect revealed by matrix stiffening leads to long-distance cell-cell communication and alignment.

Matrix-mediated mechanical coupling results in changes in cell morphology. Natan et al. observed that fibroblasts encapsulated in 3D fibrin matrices adjusted their morphology once the matrix deformation (band coupling) between two neighboring cells was formed [115]. In time, almost all cells were mechanically connected (Fig. 3b) and presented elongations and protrusions; while isolated cells remained relatively rounded (Fig. 3c, refer to the original article for the complete analysis). The results implicate again the dynamic reciprocity between cells and the matrix: Cells remodel the surrounding matrix physically to form fiber bands, driving their biological activities, and simultaneously, the densified matrix fibers promote the spreading of cells.

**Cells affecting the matrix.** In the study of Han et al., mentioned in the previous section, the authors also used microrheology to measure the nonlinear stiffening of fibrin matrices induced by single cells [34] (Fig. 3d). When human breast cancer cells were seeded in a collagen gel or Matrigel, and when HUVECs were seeded in a fibrin gel, the local stiffness correlated with the distance to the cell in the principle contraction direction. The results clearly imply that cells can generate large extended stiffness gradients in many biopolymer matrices.

Janssen et al. demonstrated that fibroblasts stiffen fibrin gels actively in a 3D culture context [113]. Cell imaging experiments in the same matrices revealed that this stiffening effect was established when the cells spread, thereby applying traction forces on the fibrin fibers (Fig. 3e). Nonlinear rheology experiments of the matrix with and without cells, indeed, confirmed that the observed stiffening of the fibrin network was induced by cell contraction (Fig. 3f) and not simply the result of the cells acting as cross-linkers (treatment with myosin-inhibitor blebbistatin prevented gel stiffening). Thus, the 3D fibrin network is actively stiffened by the contractile stress generated through the fibroblasts in the network.

Similar to collagen matrices, the stiffening of fibrin gels is associated with the aligning of fibrin fibers. Matsumoto et al. studied the effect of fiber alignment of fibrin matrices on cells (myoblasts and HUVECs) [114]. Various continuous, uniaxial static strains were applied to cell-gel constructs, and the cells in the fibrin gel aligned parallel to the strain direction. The proliferation of myoblasts (Fig. 3g) and lumen formation by HUVECS (Fig. 3h) were also responsive to the applied external strain. This system enables the *in vitro* reproduction of 3D cell alignment replicating biological tissue patterns. From a mechanical point of view, the matrix stiffening induced by fiber alignment potentially plays a role in guiding cell behavior.

Likewise, pairs of cells or cell aggregates can also induce long-distance bands of deformed fibers in fibrin matrices. Natan et al. embedded fibroblasts in fibrin gels, and monitored band formation by real-time confocal microscopy [115]. Quantitative analysis of band formation revealed an increase in fiber density and alignment between pairs of cells. The authors confirmed that the observed matrix remodeling was mainly induced by intracellular actin-myosin contraction (Fig. 3 i & j). Moreover, computational modeling suggested that the direction of cellular forces can be applied in a wide range of angles relative to a neighboring cell. In all, the reports using either collagen or fibrin matrices show a consensus that long-range mechanical coupling between cells is a universal mechanism in natural ECM polymer matrices.

## 5 Synthetic PIC hydrogel

### 5.1 About the material

A current dilemma in the field of matrix mechanobiology is that it is virtually impossible to study the role of nonlinear mechanics systematically due to a lack of suitable materials that allow for independent control of architectural and mechanical parameters. In 2013, a synthetic fibrous hydrogel based on PIC was introduced, which combined the porous architecture and mechanics of collagen and fibrin gels with the versatility of synthetic gels [[116], [117], [118]]. Indeed, the molecular structure is highly flexible: the core dipeptide (by default a *d* and *l* alanine) that is responsible for the stiffness of the chain [85], can be expanded to give stiffer chains [117] and the attached short ethylene glycol tail can be changed to vary the gelation temperature between 5 and 60 °C [87]. Copolymerization of different monomers allows the introduction of functional groups in the main chain, without loss of properties [90,91]. Post-functionalization approaches with these functional groups have been used to conjugate larger moieties, including peptides [74,119], and antibodies [120].

The customizability of PIC gels extends to the linear and the nonlinear mechanical properties. Similar to gels of collagen and fibrin, PIC gels possess a clear strain-stiffening regime, accessed at relatively low stresses or strains. The ‘static’ stiffness, critical stress, and stiffening index are tuned by polymer concentration, but also by the polymer length, which is experimentally easy to modify. Modifying polymer length offers a unique approach to manipulate stiffness without changing the gel architecture. More specifically, an increase in polymer concentration or contour length both result in a stiffer, less stress-sensitive (lower critical stress), and less responsive (lower stiffening index) gel, albeit to a different extent. Notably, the fibrous structure of the gels does not change with the polymer length, indicating that only changes in polymer concentration can result in a major difference in network structure [89].

The PIC gel is particularly suited as a 3D cell culture matrix, not only due to the architectural similarities with biological gels but also because of the thermoreversibility of the gelation process: cooling below the gelation temperature transforms the gel back into a polymer solution, which streamlines cell or cell construct harvesting [74,116]. The gels are fully biocompatible, also in *in vivo* settings. Beyond their applications as *in vitro* cell culture matrices, PIC gels are considered as artificial cytoskeleton models [121], for 3D printing [122], in antimicrobial assays [[123], [124], [125]], and for wound healing [[126], [127], [128]], immunological [129] and periodontal applications [130,131].

### 5.2 Mechanoreciprocity between cells and PIC matrices

As a relatively young material, the number of studies using PIC is still limited compared to the body of research using its natural counterparts. Nevertheless, the promising potential of this synthetic fibrous matrix has been increasingly revealed. Here, we discuss a few key representative works highlighting the versatile applications of PIC in *in vitro* cell studies. We note once again that PIC has also been utilized for a number of applications in many different fields, however, we will not go into detail about these uses and focus on the use of PIC as *in vitro* models to investigate cell-matrix mechanoreciprocity.

**Fig. 4:**
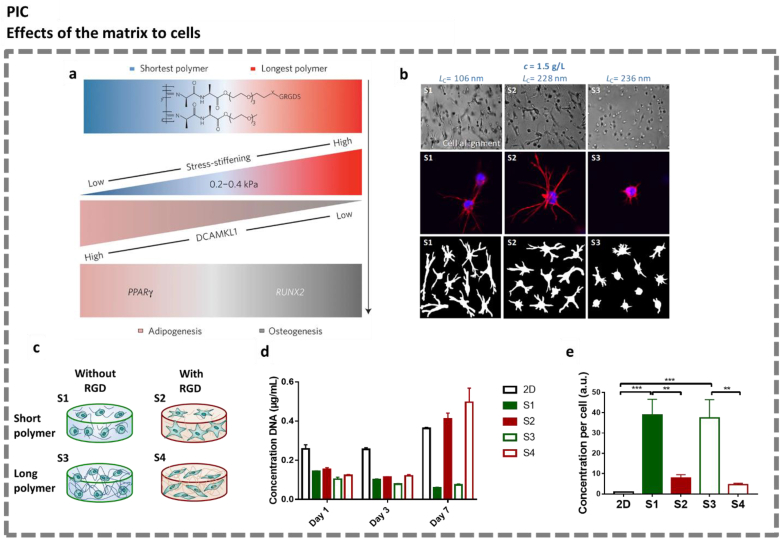
**Mechanoreciprocity between cells and PIC matrices. a.** Schematic showing the overall trends of the mechanisms by which the nonlinear mechanics of PIC regulates hMSCs commitment and differentiation towards adipogenesis and osteogenesis via modulation of the protein expression of DCAMKL1. Figure adapted from Das et al. [119]. **b.** . Influence of nonlinear mechanics of the PIC matrix on the spreading of hASCs. Top row: Representative bright-field images of hASCs. Middle row: Representative fluorescence images of hASCs; nuclei are stained with DAPI (in blue), and F-actin is stained using Texas Red Phalloidin (in red). Bottom row: Cell outlines of ten representative cells. All images were taken 3 days after cell encapsulation; Figure adapted from Liu et al. [74] **c-e.** Stem cell secretome is influenced by matrix properties. **c.** Schematic depicting properties of PIC matrices S1–S4. **d.** The quantification of DNA in cells cultured in different conditions. **e.** Expression of IL-10 per cell in different culture conditions evaluated by ELISA. Figure adapted from Liu et al. [132]. Reproduced with permission.

**The matrix driving cell behavior.** While Bruekers et al. investigated the morphology and differentiation of stem cells on top of hybrid fibrin/PIC matrices [133], Das et al. demonstrated the influence of nonlinear mechanics on stem cell differentiation using soft PIC matrices with similar stiffness but different critical stresses, in a 3D context [119]. Using Western blots and RT-PCR, they observed that the commitment of adipose-derived stem cells (hASCs) could be switched from adipogenesis to osteogenesis by changing the onset of stress stiffening (through changes in polymer length). These results suggest, for the first time, that a stress-stiffening-mediated mechanotransduction pathway governs stem cell differentiation via microtubule activities (Fig. 4a).

From a biophysical perspective, Liu et al. discussed how the nonlinear mechanics of PIC matrices affects the spreading of stem cells [74] (Fig. 4b). PIC gels with the same concentration but different critical stresses were prepared. The authors found that encapsulated hASCs spread out more in gels with lower critical stresses, which implies that the cells show stronger spreading in gels with a stronger stiffening response to external stress. The results highlight the role of the nonlinear mechanics of the extracellular matrix (and of its synthetic mimics) in the regulation of cell functions.

To further investigate the regenerative potential of 3D stem cell culture, Liu et al. studied the paracrine functions of hASCs in response to matrix properties [132] (Fig. 4c–e). The stem cells were encapsulated in PIC matrices with different mechanics and biofunctionalization, all at the same polymer concentration (Fig. 4c). Proliferation assays proved that cell adhesiveness over nonlinear mechanics dominated cell growth, namely, cells only proliferate when the 3D matrix supports their adhesion (Fig. 3d). Multiplex assays and ELISA revealed that cells that could not interact mechanically with the matrix produced increased levels of IL-10 (Fig. 4e). Conditioned medium from 3D matrices induced significantly faster wound closure compared with that from control 2D cultures using tissue culture polystyrene. Notably, the nonlinear mechanics of the gels impacts the morphology of the cells but not their secretome. These results emphasize the versatility and potential of PIC hydrogels to serve as a multitool to uncover the regenerative potential of stem cell secretome.

Zhang et al. [134] and Ye et al. [135] showed the potential of PIC matrices for organoid culture. They showed that for a number of different organoids (liver, mammary gland, prostate) tailoring of the PIC matrix properties can be used to tune the organoids characteristics. In a separate contribution, morphogenesis and mechanotransduction of Madin‐Darby Canine Kidney (MDCK) cells in hybrid PIC/Matrigel matrices were discussed [24]. Although the authors confirmed the strain-stiffening properties of the PIC and composite matrices, they did not directly investigate the role of the nonlinear mechanics in these studies.

**Cells affecting the matrix: to be explored.** Fluorescence imaging has revealed PIC as a biomimetic fibrous network at the micron scale [89], however, reports on the cell-mediated matrix stiffening at the macroscopic scale and fiber remodeling at the micron scale are still pending. Nevertheless, some hints are implicating the existence of integrin-mediated mechanical communications through the fiber network between cells. For instance, aligning stem cells have been observed in a PIC-RGD matrix [74], as well as macroscopic contraction after long-term culture of contractile cells. Quantitative studies are required to unveil the cellular impacts on the PIC matrix.

The investigation on the long-term degradability of PIC is still in process, although several *in vivo* reports find that PIC gels do not degrade readily within several weeks to months [126,127]. This raises the question: can cells remodel the PIC matrix to initiate the typical responses of its natural collagen or fibrin counterparts? Recent work based on alginate hydrogels confirmed that in a physically crosslinked polymer network with sufficient mechanical plasticity, a protease-free migration mode exists where cells do not need enzymatic degradation to spread and migrate [55]. For PIC-based synthetic matrices one may expect an analogous (bio)chemical-independent remodeling mechanism (also called physical remodeling of ECM), however, supporting experimental evidence is needed.

## 6 Outlook

**Scheme 2:**
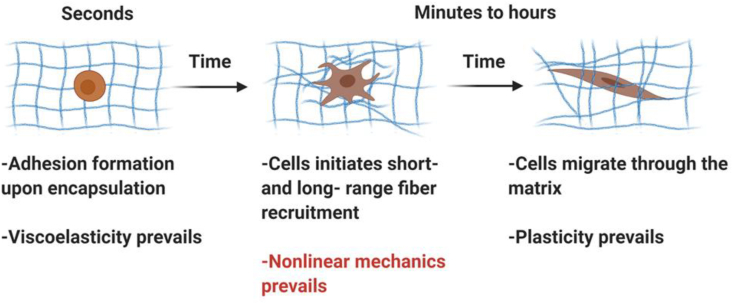
**Viscoelasticity, nonlinear mechanics, and plasticity with time in 3D fibrous matrices.** Biophysical changes, such as cell-mediated fiber rearrangement and fiber-induced spreading and migration of contractile are commonly observed in fibrous biological and biomimetic gels. Different types of mechanical properties dominate at different time scales, possibly with mutual interactions [56]. Note that the extent and rate of cell development and matrix remodeling will strongly depend on the physical properties of the matrix, cell-matrix interactions as well as soluble cues inside the matrix.

As a practical summary, Table 1 provides a comparison between key parameters of collagen, fibrin, and PIC-based matrices. We included Matrigel as the ‘golden standard’ in the field, although from the table it is clear that its architecture and mechanical properties poorly match with the fibrous matrices. The table lacks a comprehensive overview of viscoelastic and plasticity data on the matrices, a topic that is currently seeing increasing interest [36,75,136]. It is broadly recognized that the mechanical properties of the natural ECM are not constant in time and that different mechanical mechanisms may dominate at specific cellular time scales. For instance, stress relaxation is considered to be important, particularly at shorter time scales (from tens to hundreds of seconds) [75], once cells form adhesion with the surrounding fibers. Nonlinear mechanics, i.e. strain stiffening (the focus of this review) is a strain-dependent but not time-dependent characteristic and often requires a longer time scale to be detected under cellular contraction (from hours to days), as cells apply contractile forces and initiate physical matrix remodeling; plasticity has been proved to show up at a longer time scale than stress relaxation (from minutes to hours) [36,137], typically detected as cells migrate through the matrix (Scheme 2).

**Table 1:** Comparison between common polymer matrices as strain-stiffening models.

	Collagen	Fibrin	PIC	Matrigel
Origin	animal	animal	synthetic	animal
Controlled composition	+	+	+	–
Gelation method	pH	enzymatic	temperature	temperature
Fibrous structure	+	+	+	–
Typical pore Size	micron [138]	micron [138]	micron [89]	micron [139]
Degradability	+	+	–	+
Typical concentration (mg/ml)	0.1–3	0.1–8 [96,113]	0.1–3 [74,89,119]	~3–4 [58]
Typical stiffness (Pa)	<10^3^ [58],[a]	<10^3^ [96]	<10^3^ [74]	<10^3^ [30]
Nonlinear mechanics [b]	++	+	+++	+
Factors determining *m*[c]	polymerization temperature [140]	polymerization conditions [96]	polymer length or concentration [74,118]	unknown
Cell adhesion sequence	GPOGPO, GFOGER, RGD [141],[d]	mainly RGD [142]	customizable (click chemistry)	RGD, PDSGR, YIGSR, IKVAV [143],[d]

[a] Without extensive chemical crosslinking.

[b] The number of + indicates the relative achievable degree of stiffening (as indicated in Fig. 1e).

[c] *m*: stiffening index.

[d] GPOGPO = glycine-proline–hydroxyproline-glycine-proline–hydroxyproline; GFOGER = glycine-phenylalanine-hydroxyproline-glycine-glutamate-arginine; RGD = arginine-glycine-asparagine; PDSGR = proline-asparagine-serine-glycine-arginine; YIGSR = tyrosine-isoleucine-glycine-serine-arginine; IKVAV = isoleucine-lysine-valine-alanine-valine.

Is mechanoreciprocity generalizable or material-specific? This is a central question in discussions in the field of tissue biophysics. As we know, mechanoreciprocity *in vivo* takes place between cells and a mixture of fibrous and nonfibrous biopolymers. To disentangle the complex interactions, simplified polymer matrices are used. At this stage, in a broad sense, evidence points out that cell-matrix mechanoreciprocity can be generalized for fibrous matrices with nonlinear mechanics when compared with non-fibrous matrices such as dense basement membranes [94]. For instance, cell-induced ECM fiber recruitment, stress propagation, and heterogeneous stiffening are seen in both collagen and fibrin. At the same time, in a narrower sense, there exist many known (and unknown) polymer-specific differences as well, caused by different mechanics, architecture, and biochemical composition of matrices. Again, the answer to the question is not fully clear yet, awaiting more investigation using consistent methodologies and proper material controls.

We stress the importance of fully characterizing all aspects of the mechanical properties of matrices and we believe that we will find that ultimately, the goal is not to develop a matrix that mimics the ECM best, but rather a matrix that has all its properties optimized for a desired *in vitro* application. To be able to streamline the optimization process, independently controllable properties are highly beneficial, which should include, besides the full range of mechanical properties, the hierarchical assembly of fibers, and their biological interactions, e.g. density and type of cell adhesive sequences. Alternative approaches to modularly adapt matrix properties are through (selective) crosslinking [116] or generation of mixed/linked hybrids or composites [98,121].

Advances in tissue engineering and regenerative medicine depend heavily on the development of suitable materials. Since the pioneering work from Engler et al. [144], which can be seen as the first-generation design principle, it is universally accepted that static mechanics of biomaterials should be taken into consideration in tissue construction. Responsive or dynamic mechanics, which refers to all mechanical properties, linear, nonlinear and viscoelastic, that change in response to external stimuli, including cellular contraction remain awfully difficult to engineer. To promote the second-generation design principle which highlights the dynamic mechanics of ECM, more studies are urgently required, which not only demonstrate the biophysical responses of cells but also the biological feedback at all levels from molecules to tissues. Key questions in this research will include:

1. Do the origins of nonlinearity matter in the context of cell mechanosensing and the mechanical forces exerted by the cells?
2. Does (cell-induced) adaptive mechanics trigger different cellular signaling and transcription pathways, as well as a difference in the epigenome [75] compared with 2D and 3D linear materials? If yes, why and how?
3. How do tissues coordinate different types of dynamic mechanics? And what material, like hydrogels, (electro)spun or extruded fibers [145] or composites of different materials can accurately capture the architecture and mechanics of native ECM?
4. What are the exact functions of nonlinear (responsive) mechanics *in vivo*? Why do tissues evolve to possess these properties?

A technical challenge is the choice of suitable materials that are able to provide answers. Biological matrices prepared from natural polymers can resemble the *in vivo* microenvironment but lack the tunability of individual properties, which makes it challenging to decipher the impact of individual parameters. Synthetic matrices prepared from conventional synthetic polymers, however, often lack biomimicry in some crucial aspects. New-generation synthetic materials, such as PIC or the recently published bolaamphiphiles [146], offer an almost endless amount of possibilities thanks to the combination of a fibrous architecture (with nonlinear mechanics), customizable functional sites, and a high degree of freedom in individual parameter control. Evidence has shown that many other man-made synthetic fibrillar gels have the potential to possess the strain stiffening nature [76], and we foresee an expansion of promising synthetic materials in the near future. One of the downsides of the synthetic matrices is their lack of adhesion factors is commonly compensated through the addition of ECM protein mimicking peptides, which provide a good first-order alternative but may not capture the finer details of the cell-gel interaction.

Although this manuscript mainly discusses biological or synthetic minimalist models that primarily replicate the structural components of the ECM, we underline that simplified *in vitro* cell-matrix models often lack the compositional and structural complexity of tissues and organs *in vivo*. As an example, the high cell volume fraction greatly influences the mechanics of tissues [51]. We refer the interested reader to reviews on tissue-specific mechanobiology of bone [147], tendon [148], cardiac [149] and vascular [150] tissues. To engineer constructs better mimicking complex 3D biological structures, techniques such as bioprinting have emerged as an attractive approach [151]. For instance, tubular biological materials [152] and mineralized bone scaffolds with custom shapes [153] can be readily realized by combing various printing methods but remain challenging to fabricate by traditional approaches. Interestingly, bioprinting benefits from another nonlinear mechanical effect: shear thinning, where the modulus drops with increased strain. Recent publications showed the first examples of synthetic hydrogels (nonfibrous) that combine shear thinning with strain stiffening properties [71,72]. In the majority of bioprinting contributions, however, researchers have not elaborated on the nonlinear mechanics of the matrix (ink) and its role in regulating the functions of the printed tissues. In other words, studies on matrix properties and tissue engineering are yet to be integrated. Filling the gap between mechanisms and the applications will be a giant leap for biophysicists and tissue engineers towards the ultimate goal, the fabrication of functional living materials. Last but not least, appropriate techniques together with computational models will also facilitate the process to disentangle the continuous cell-matrix interactions in 3D, serving as forceful tools [154,155].

## 7 Concluding remarks

Well-defined *in vitro* models are necessary to unravel the complex reciprocal interactions between the extracellular matrix and cells, which ultimately are required, to develop engineering strategies to drive cell behavior in any desired direction. To realize an *in vivo*-like response, we should first focus on the use of 3D matrices with architecture and mechanical properties that resemble native tissues. Biology in 3D matrices prescribes a sufficiently porous (and cleavable) network architecture to allow cell proliferation and migration. The mechanical properties are more complex as the stiffness of the natural ECM changes considerably upon the contractile activity of embedded cells, particularly in the cellular microenvironment. To date, the number of studies that investigate the effects of this stress-stiffening feature is still limited; the majority of studies still only consider the ‘static’ stiffness.

So far, the best performing models that include a more dynamic global and local stiffness are based on the fibrous ECM proteins collagen and fibrin, despite their limitations in tunability. Synthetic matrices that intrinsically possess enormous tailorability often lack the architecture and/or the mechanical properties that match with the biological materials. This review summarizes the current progress in understating the role of nonlinear mechanics in cell-matrix mechanoreciprocity and substantiates the possibilities that traditional and novel polymer gels can offer.

## CRediT authorship contribution statement

**Kaizheng Liu:** Writing – original draft, Writing – review & editing, wrote the original draft, edited the draft until its final form. **Maury Wiendels:** Writing – original draft, wrote the original draft. **Hongbo Yuan:** Writing – review & editing, edited the draft until its final form. **Changshun Ruan:** Writing – review & editing, edited the draft until its final form. **Paul H.J. Kouwer:** Writing – review & editing, Supervision, edited the draft until its final form. supervised the project.

## Declaration of competing interest

The authors declare no conflict of interest.
